# First record of the ectoparasitic nematode *Amplimerlinius macrurus* (Nematoda: Tylenchida) on the perennial grass *Miscanthus* × *giganteus* (Angiosperms: Poaceae) in Ukraine

**DOI:** 10.21307/jofnem-2021-024

**Published:** 2021-03-06

**Authors:** Tatyana Stefanovska, Andrzej Skwiercz, Łukasz Flis, Valentina Pidlisnyuk, Miloslav Zouhar

**Affiliations:** 1 Department of Entomology, National University of Life and Environmental Sciences, Kiev, Kyiv; 2 Department of Plant Protection, Research Institute of Horticulture, Skierniewice, Poland; 3 Museum and Institute of Zoology, Polish Academy of Sciences, Warsaw, Poland; 4 Department of Environmental Chemistry and Technology, Jan Evangelista Purkyne University, Usti nad Labem, Czech Republic; 5 Department of Plant Protection, Czech University of Life Sciences, Prague, Czech Republic

**Keywords:** Plant-parasitic nematode, Taxonomy, Morphological description, Genetic analysis, Ukraine

## Abstract

The growing interest in biomass production of *Miscanthus* × *giganteus* (*M × g*) (Poaceae) on agricultural and marginal lands has prompted researches to identify plant pathogens and diseases affecting this crop which has a great potential for production of biofuels and different bioproducts. A soil survey of nematodes in the *M × g* rhizosphere and a report on the collection of the plant-parasitic nematode *Amplimerlinius macrurus* (Belonolaimidae) were accomplished in two locations in Ukraine. It is known that this family of nematodes can reduce the root system and biomass of Poaceae family plants. Both molecular and morphological characters were used to identify the nematodes; measurements and photomicrographs of the species were presented. This is the first documentation and description of *A. macrurus* in Ukraine to the best of our knowledge. Further investigation is underway to confirm the pathogenicity of this species on perennial grasses plantations.

*Miscanthus* × *giganteus* J. M. Greef, Deuter ex Hodk., Renvoize (Angiospermae: Poaceae) (*M × g*) is a rhizomatous, lignocellulose-rich perennial grass grown worldwide as a biofuel crop and a source of different bio-based products (Clifton-Brown, 2017; Cosentino et al., 2018; Lewandowski et al., 2018). This plant is the most common second-generation biofuel crop for commercial production in Ukraine due to its rapid growth and high yields in agricultural and marginal/contaminated soils of various anthropogenic origins (Kharytonov et al., 2019; Pidlisnyuk et al., 2020). Stefanovska et al. (2017) found several herbivorous insects associated with the cultivation of *M × g* being grown in the plantation as a source of bioenergy. Plant-parasitic nematodes may also affect *M × g* yields (Mekete et al., 2009, 2011). Different agronomic practices affect *M × g* yield and nematode communities, the latest can be used as a bioindication of the phytoremediation process (Almasary et al., 2020). Earlier, a soil survey on identifying plant-parasitic nematodes associated with the cultivation of *M × g* was conducted in eight locations in Ukraine (Stefanovska et al., 2020) in order to assess the potential for nematodes to reduce yield. During the survey *Amplimerlinius macrurus* (Goodey, 1932) was detected in the soil under *M × g* at two of eight locations: those locations were Dolyna (site was located within city) and Grytzyv (village is located in Khmelnitska region).

The history of the species knowledge may serve as an example of confuse state of nomenclature. It started in 1914 with a first note on *Aphelenchus dubius* spez. nov. by Steiner from Switzerland (Steiner, 1914) and was gone through several name changes and genus transfers. In 1932, Goodey redescribed the species and named it as *Anguillulina macrura*, which was a feeder in the cortical cell layer of the roots of the grass *Lolium perenne* L. (Goodey, 1943). The next name change was made in 1936 in the new genus *Tylenchorhynchus macrurus* (Filipjev, 1936) and redescribed by Wallace and Greet (1964). Finally, the last replacement of the species was done by Siddiqii (1976), while it was renamed *Amplimerlinius macrurus* (Filipjev, 1936; Goodey, 1932). The problem with taxonomic uncertainty was shown by study of Siddiqii and Klingler (1980) which collected specimens of *A. dubius.* Steiner in the type locality of *A. dubius* near Thalwil (Switzerland) proposed *Amplimerlinius dubius* comb. n. for *A. dubius* (Steiner, 1914).

*A. macrurus* was found in the soil under different land use and cover types, i.e.: crops, meadows, pastures, grasslands, forest, fruit, nut, and olive orchards. The species was reported by researchers from the United States (Norton et al., 1984), as *A. dubius* (Steiner, 1914) and different parts of Europe (Háněl and Čerevková, 2006; Lamberti and Vovlas, 1993; Renčo, 2013), Caucasus, i.e.: Armenia (Poghossian, 1979) and Azerbaijan (Niknam et al., 2008), Asia, i.e.: Tajikistan (Ivanova, 1982); countries in the Middle East (Bahmani et al., 2013; Ghaderi et al., 2017; Hashim, 1982, 1983; Panahandeh and Pourjam, 2014; Stefan et al., 1985). Specifically, Ghaderi and Karegar (2014) and Ghaderi et al. (2014) provided lists of different field crops and *A. macrurus* was associated with Iran. It is presented in the latest updated parasitic annotated checklist of parasitic nematodes in Germany (Sturhan, 2014) and Belgium (Bert et al., 2003).

*A. macrurus* is associated with many cultivated crops and grasses (Bert et al., 2003; Kheiri et al., 2002; Navas and Talavera, 2002; Winiszewska et al., 2012). However, it was not previously recorded at the energy crop like *M × g.* The objective of this study was to describe two populations of *A. macrurus* associated with *M × g* via morphological, morphometric, and molecular approaches.

## Materials and methods

The soil samples were collected in 2017 from the rhizosphere of two-year *M × g* stands located in Dolyna city, Ivano-Frankivsk region, Ukraine (49.15°N, 24.37°E) on Glayic Cambisols: and in Grytzyv village in the vicinity of Shepetivka, Khmelnytskyi region (49 98°N, 21 36°E) from the rhizosphere of five-year *M × g* stands on Chernozem. A sampling of nematodes was done randomly through late August-early October.

The rapid centrifugation-flotation method (Szczygieł, 1971) was used for extracting nematodes from the soil samples. Isolated nematodes were killed and fixed, then passed through a graded series of glycerol-ethanol solutions, followed by storage in anhydrous glycerol on permanent slides (Seinhorst, 1959). In total, 47 individuals from the sample were identified to the species level morphologically via a Leica biological microscope using the keys of Andrássy (2007), Brzeski (1998), and Ghaderi et al. (2014).

In order to confirm a morphological identification, three putative *A. macrurus* specimens were fixed in a DESS solution (Yoder et al., 2006) for genetic analysis. After washing with sterilized milli-Q water, selected individual nematodes were transferred to separate 0.2 ml polymerase chain reaction (PCR) tubes containing 25 μl sterile water, then lysed for DNA extraction according to the procedure described by Holterman et al. (2006). The obtained single nematode lysate (crude DNA extract) was used as a DNA template for a PCR reaction or stored at −20°C. 18S rDNA was amplified in two overlapping fragments using the following primer combinations: 988F with 1912R and 1813F combined with 2646R (Holterman et al., 2006). Amplification of the partial 28S rDNA sequence was obtained using the D2A and D3B primers (Nunn, 1992). The 18S and 28S rDNA regions were sequenced by the Sanger method on the ABI 3500L genetic analyzer (Applied Biosystems, Foster City, CA, USA). The sequences reported in this study were deposited in GenBank under the following accession numbers: MK952146 for 18S rDNA and MK951999 for 28S rDNA.

## Results and discussion

**Figure 1:**
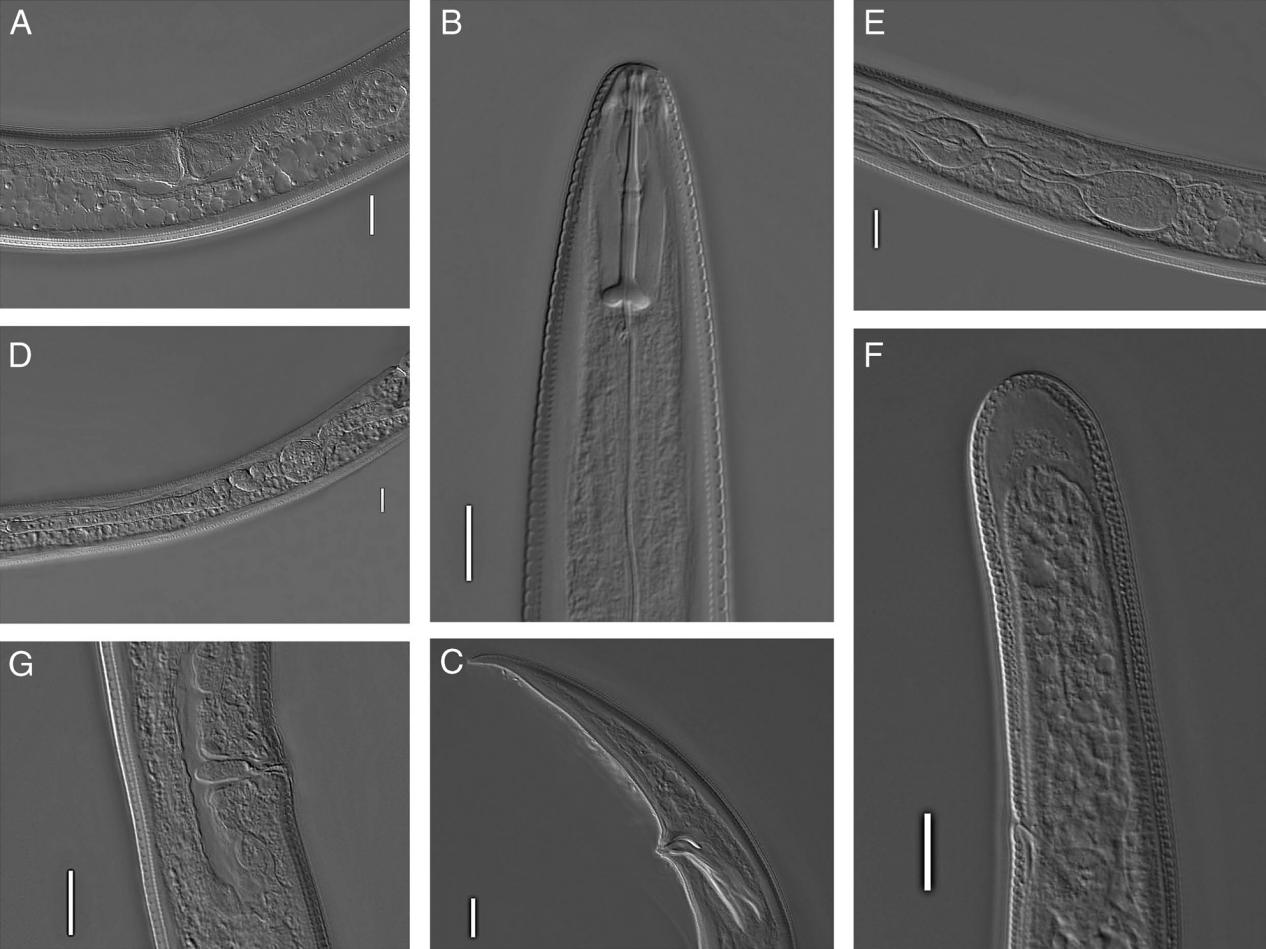
Photomicrographs of *Amplimerlinius. macrurus* adults. A. Part of female reproductive system with spermatheca filled with sperm; B. Anterior part of female; C. Male, posterior end of body with arch like shaped gubernaculums; D. Female reproductive system; E. Pharyngeal system; F. Female tail; G. Vulva. (scale bars = 10 μm).

*A. macrurus* was collected in two locations representing population densities 12 to 110 individuals in 100 g of samples of two soil types: *M × g* stands in Dolyna (measurements for 20 females and 10 males), and *M × g* stands in Grytzyv (measurements for 15 females and two males). The original photomicrographs of this species are presented in Figure 1 and morphometrics are given in Table 1. The photomicrographs of the collected specimens showed that the continuous head region and distinct clavate tail shape provided an evidence that it was *A. macrurus*.

**Table 1.** Morphometrics of *Amplimerlinius macrurus* from two locations (Dolyna, Grytzyv) in Ukraine.

	Dolyna		Grytzyv		Total	
Character	Females	Males	Females	Males	Females	Males
*n*	20	10	15	2	35	17
*L*	823 ± 11 (805-840) 1.4	746 ± 6.0 (730-755) 0.8	786 ± 18 (755–805) 2.3	718 ± 2.8 (716-720)	808 ± 23 (755-840) 2.8	742 ± 12.6 (716-755) 1.7
*a*	27.1 ± 1.1 (25.5-28.5) 4.1	28.1 ± 0.4 (27.6-28.6) 0.4	25.9 ± 0.5 (25.4-26.8) 1.9	28.5 ± 0.1 (28.4-28.6)	26.6 ± 1.0 (25.4-28.5) 4.0	28.2 ± 0.4 (27.6-28.6) 1.4
*b*	5.4 ± 0.1 (5.3-5.4) 1.4	4.6 ± 0.1 (4.4-4.8) 2.8	4.7 ± 0.2 (4.5-5.0) 4.2	4.9 ± 0.1 (4.8-4.9)	5.1 ± 0.3 (4.5-5.4) 7.2	4.7 ± 0.1 (4.4-4.9) 3.1
*c*	14.2 ± 0.8 (12.8-15.4) 5.9	12.7 ± 0.3 (12.2-13.2) 2.5	15.5 ± 0.7 (14.2-16.6.) 5.2	12.7 ± 0.2 (12.5-12.8)	14.8 ± 1.0 (12.8-16.6) 6.7	12.7 ± 0.3 (12.2-13.2) 2.3
*c′*	3.0 ± 0.2 (2.6-3.4) 9.2	3.4 ± 0.1 (3.3-3.5) 9.2	2.7 ± 0.2 (2.7-2.9) 8.0	3.4 ± 0.1 (3.3-3.4)	2.9 ± 0.2 (2.6-3.4) 10.0	3.4 ± 0.1 (3.3-3.5) 2.6
*V*	54.5 ± 7.6 (50-59) 13.9	–	53.7 ± 1.2 (52-56) 2.8	–	54.1 ± 5.7 (50-59) 10.6	–
Stylet length	26.8 ± 1.4 (25-28) 5.2	26.2 ± 1.2 (25-28) 4.6	26.8 ± 0.8 (25-29) 3.2	26.5 ± 0.7 (26-27)	26.8 ± 1.2 (25-29) 3.2	26.3 ± 1.1 (25-28) 4.3
Excretory pore	115.8 ± 3.4 (110-120) 2.9	112.2 ± 1.8 (110-115) 1.6	121.2 ± 3.6 (116-124) 2.9	121 ± 1.4 (120-122)	118.1 ± 4.4 (110-124) 3.7	113.7 ± 3.8 (110-122) 3.4
Hemizonid length	3.5 ± 0.1 (3.5-3.6) 1.4	–	3.6 ± 0.1 (3.5-3.8) 2.7	–	3.6 ± 0.1 (3.5-3.8) 2.7	–
Body width	33.4 ± 1.2 (32-36) 3.5	–	31.4 ± 1.2 (30-34) 3.9	–	32.5 ± 1.6 (30-36) 4.9	–
Tail annuli	52.6 ± 1.4 (50-54) 2.7	–	56.9 ± 3.9 (48-62) 7	–	54.4 ± 3.5 (48-62) 6.4	–
Tail length	58.5 ± 5.4 (49-68) 9.2	–	56.4 ± 1.7 (54-58) 3.0	–	57.6 ± 4.3 (49-68) 3.6	–
Phasmids on tail /%	46.0 ± 1.9 (42-48) 4.2	–	45.2 ± 1.1 (44-47) 2.7	–	45.6 ± 1.6 (42-48) 3.7	–
Hyaline/tail %	19.6 ± 1.3 (18-22) 7.0	–	20.6 ± 1.1 (18-22) 5.7	–	20.0 ± 1.3 (18-22) 6.8	–
Gubernaculum	–	12.1 ± 1.3 (10-14) 11.3	–	11 ± 1.4 (10-12)	–	11.9 ± 1.4 (10-14) 11.6
Spicule length	–	30.7 ± 2.4 (28-34) 7.8	–	32.5 ± 0.4 (32-33)	–	31 ± 2.3 (28-34) 7.4

**Note:** All measurements are in µm and in the form: mean  ±  s.d. (range) CV %.

### Description of Ukrainian specimens

Female body C-shaped: *L* = 805 to 840 µm (Grytzyv), vs. 755 to 805 µm (Dolyna). Cuticle annulated prominently, 1.1 to 1.2 µm wide at mid-body. Lateral field 10 to 13 µm wide, 27 to 32% of body width, with six lines. Head conoid with six annuli and flattened apex, continuous with body contour. Stylet length 25 to 29 µm, well developed; shaft equal to conus, *m* = 49 to 50, knobs 5.7 to 6.0 µm across, slightly sloping backward. Orifice of dorsal pharyngeal gland 3.4 to 3.8 µm from the base of stylet. Hemizonid 3.5 to 3.8 µm long, anterior to excretory pore, 110 to 124 µm from anterior body end. Median bulb slightly elongated 20-24 µm × 12-14 µm, with prominent valve plates. Nerve ring 105 to 110 µm from the anterior end. Basal bulb cylindrical, 30 to 35 µm long and 18 to 20 µm wide, length/width ratio 1.5 to 2.0, offset from the intestine, cardia oval, prominent. Head-to-vulva distance 410 to 455 µm. V ratio in both populations 50 to 59%. Vulva flush with the body surface. Some individuals with vulval flap, 1 to 15 µm wide and 2.5 to 3 µm long. Vagina about half body width, not sclerotized. Spermatheca rounded, with sperm. Ovaries outstretched, each with a single row of oocytes. Rectum about half of anal body width. Head-anus distance 765 to 780 µm (Grytzyv) vs. 690 to 765 µm (Dolyna). Tail clavate, with 50 to 54 (Grytzyv) vs. 48 to 62 (Dolyna) annuli at ventral side, tail terminus annulated. Tail length: 49 to 68 µm (Grytzyv) vs. 54 to 58 µm (Dolyna). The ratio *c* = 12.8 to 15.4 (Grytzyv) vs. 14.2 to 16.6 (Dolyna), *c′* = 2.6 to 3.4 (Grytzyv) vs. 2.7 to 2.9 (Dolyna). Terminal hyaline region 8 to 12 µm, occupying 18 to 22% of tail length in both populations. Phasmids 2 to 3 µm in diameter, 42 to 48% of tail length (Dolina) vs 44 to 47 (Grytzyv) in both populations.

Male body C-shaped: *L* = 730 to 755 µm (Grytzyv) vs.716 to 720 µm (Dolyna). Head, stylet, pharynx, lateral fields, and annuli: similar to females. Spicule length: 28 to 34 µm (Grytzyv) vs. 32 to 33 µm (Dolyna) Gubernaculum arch-like shaped, the string of arch: 10 to 14 µm. Bursa is located 5 to 8 µm anteriorly to the spicule base to the *C*-shaped thin tail end. Phasmids 40 to 44% of tail length.

The amplification of the almost full-length 18S rDNA fragment was successful (1,670 bp, MK952146). The Basic Local Alignment Search Tool (Blast) showed a 99.52% similarity to the sequence of *A. macrurus* deposited in GenBank from the Netherlands (FJ969114) (Carta et al., 2010). The 28S rDNA sequence alignment from *A. macrurus* (712 bp, MK951999) showed a 99.30% similarity to the sequence of *A. macrurus* deposited in GenBank from Iran (KX789694). Our investigation showed that the results of *A. macrurus* molecular identification corresponded to its morphological diagnosis.

*A. marcurus* was first isolated in Poland from peat soils in grasslands by Skwiercz (1989) and Brzeski (1998). Brzeski (1998) considered *A. macrurus* as a sedentary ectoparasitic grass nematode. However, the data regarding the feeding type on new perennial grasses, including *M × g*, is still lacking. The growing needs for such data are attributable to the rapid upscaling of *M × g* cultivation in Ukraine in agricultural, marginal, and deteriorated lands and increasing risk of nematode damage and adverse impact to the biomass yield. Generally, the results of the study of morphological and morphometric characters of two Ukrainian *A. macrurus* populations appeared consistent description after Wallace and Greet (1964) and Saltukoglu et al. (1976). Differential analyze of several populations described from England, after Siddiqii (1976) [S], Spain after Bello et al. (1987) [B], and Iran after Ghaderi and Karegar (2014) [G] showed wider range of the morphometric characters. The most of its indices fits well to the body length, head annuli, *a*, *b*, *c*, *V*, tail, and stylet length and gubernaculum of Ukrainian [U] populations. Some characters appeared wider or shorter in Ukrainian populations, i.e.: shorter pharynx: 128 to 142 µm [U] vs. 145 to 205 µm [S] and 160 to 180 µm [G]; head-anus distance: 600 to 780 µm [U] vs. 650 to 685 µm [G]; number of tail annuli: 48 to 62 [U] vs. 30 to 47 [B]; *c′*: 2.6 to 3.5 [U] vs. 2.2 to 3.2 [S], 2.5 to 3.1 [B], 2.1 to 3.1 [G]; MB: 48 to 57% [U] vs. 52 to 59 [S], 49 to 52 [G]; hyaline length: 18 to 22 µm [U] vs. 9 to 15 µm [S], 8 to 15 µm [G]; spicule length: 28 to 34 µm [U] vs. 31 to 40 µm [S], 31 to 38 µm [G]; excretory pore length: 110 to 124 µm [U] vs. 120 to 154 µm [G].

To the best of our knowledge, this is the first record of *A. macrurus* associated with *M × g*. The received data confirmed the occurrence of this nematode in Ukraine and extended the range of host crops for these species. Our findings provided some basic arguments for considering *A. macrurus* as a potential pest of perennial crops in Ukraine. However, for demonstration its pathogenicity and host range on *M × g* it is advisable to validate this study hypotheses in the future research.
